# A pilot study of risk-stratified cervical cancer screening

**DOI:** 10.12688/openreseurope.13398.2

**Published:** 2022-09-01

**Authors:** Jiangrong Wang, K. Miriam Elfström, Christer Borgfeldt, Joakim Dillner

**Affiliations:** 1 Department of Laboratory Medicine, Karolinska Institute, SE-141 52 Stockholm, Sweden; 2 Regional Cancer Center Stockholm Gotland, Lindhagensgatan 98, SE-102 39 Stockholm, Sweden; 3 Karolinska University Laboratory, Karolinska University Hospital, Anna Steckséns gata 49, SE-171 76 Stockholm, Sweden; 4 Department of Obstetrics & Gynecology, Skåne University Hospital, Department of Clinical Sciences, Lund University, Klinikgatan 12, SE-222 42 Lund, Sweden

**Keywords:** Human Papillomavirus, self-sampling, SMS-based screening invitations

## Abstract

**Background:** Cervical screening programs target entire populations, although it is well established that cervical cancer risks can vary >100-fold based, in particular, on the woman’s screening history. Since cervical screening switched to Human Papillomavirus (HPV) testing as the primary screening method, the risk differences are even larger as different HPV types may vary in associated cancer risk by 100 times. Furthermore, HPV infections with the most oncogenic types are declining dramatically because of HPV vaccination programs. Tailoring screening intensity based on the known cancer risk of the individual (risk-stratified screening) therefore has great potential to increase both the sensitivity and specificity. Within Horizon 2020 a major project for Risk-stratified Screening for Cervical Cancer (RISCC) has therefore been launched. We performed a pilot study of risk-stratified screening to evaluate feasibility and acceptability of offering vaginal HPV self-sampling tests to women with a higher risk of cervical cancer.

**Methods:** We identified resident women who had had either i) atypical glandular cells in screening tests during the past six years (risk >150/100,000 woman-years) or ii) abnormal screening findings above the age of 50, but without sufficient follow-up (risk >65/100,000). The women were invited, either by short message service (SMS) or physical letters, to order an HPV self-sampling kit via the study web-platform. The returned self-collected samples were tested for HPV. If positive, women were invited for clinical follow-up.

**Results:** Among 920 targeted women, 191 (21%) placed an order and 163 (18%) returned a self-collected sample. Among all tested samples, 19 (12%) were positive for hrHPV and 18 of these women attended clinical follow-up.

**Conclusions:** SMS invitations to high-risk women complemented with physical letters are feasible and result in substantial requests for kits and submission of samples. Future work will focus on improving the efficiency of the procedure and further increasing attendance.

## Introduction

With high coverage of cervical screening and vaccination against the causative agent human papillomavirus (HPV), elimination of cervical cancer is possible. WHO has launched a global strategy to accelerate the elimination of cervical cancer as a public health problem, defined as an incidence below 4 women per 100,000 women per year ^1, 2^. As the spread of the causative agent is declining, further optimization of cervical screening programs becomes even more important. Risk-stratified screening programs are increasingly discussed as a strategy for obtaining the expected benefits of screening while also containing resource use.

Cervical cancer incidence has decreased by more than 50% after the implementation of organized cervical screening, which detects and treats cervical pre-cancerous lesions ^3^.

Around 15% of the annual cervical cancer cases in Sweden have had a history of abnormal screening results, prior to the screening test that diagnosed the cancer ^4^. Sufficient follow-up and management of women with abnormal screening findings are essential to prevent the progression from precancerous lesion to invasive cervical cancer. Our previous studies identified two groups of women who have a particularly high risk of cervical cancer: women who have had atypical glandular cells (AGC) detected in cervical screening. These women had a risk of 150 per 100,000 person-years in the subsequent 6.5 years, but many of them had not had sufficient follow-up ^5^. The other group was women older than the upper age limit of organized cervical screening but who had had abnormalities after age 50 years, exhibiting risk around 60–100 per 100,000 person-years ^6^. Many of these women were not sufficiently followed-up.

Performing risk-stratified screening targeting these women is thus urgent. However, with the conventional screening, it is difficult to fit an additional strategy within the available system that summons women for sampling at defined screening stations. Offering vaginal self-sampling for HPV testing to the high-risk women is a potential solution, as women may be resident far away from a screening station - but the women can be reached by SMS and by the postal service subsequently delivering an HPV self-sampling kit. HPV self-sampling has been found to be an acceptable, effective, reliable and lower-cost method to increase screening coverage ^7, 8^. For the two identified risk groups, HPV testing is particularly beneficial, because the HPV status can effectively distinguish the risk following AGC ^9^, and may outperform the unsatisfactory cytology for post-menopausal women ^10, 11^.

We therefore performed a pilot study of risk-stratified screening to offer vaginal self-sampling HPV testing to all women who were resident in Southern Sweden and who had AGC or older aged women with previous abnormalities, but without sufficient follow-up.

## Methods

**Figure 1.**
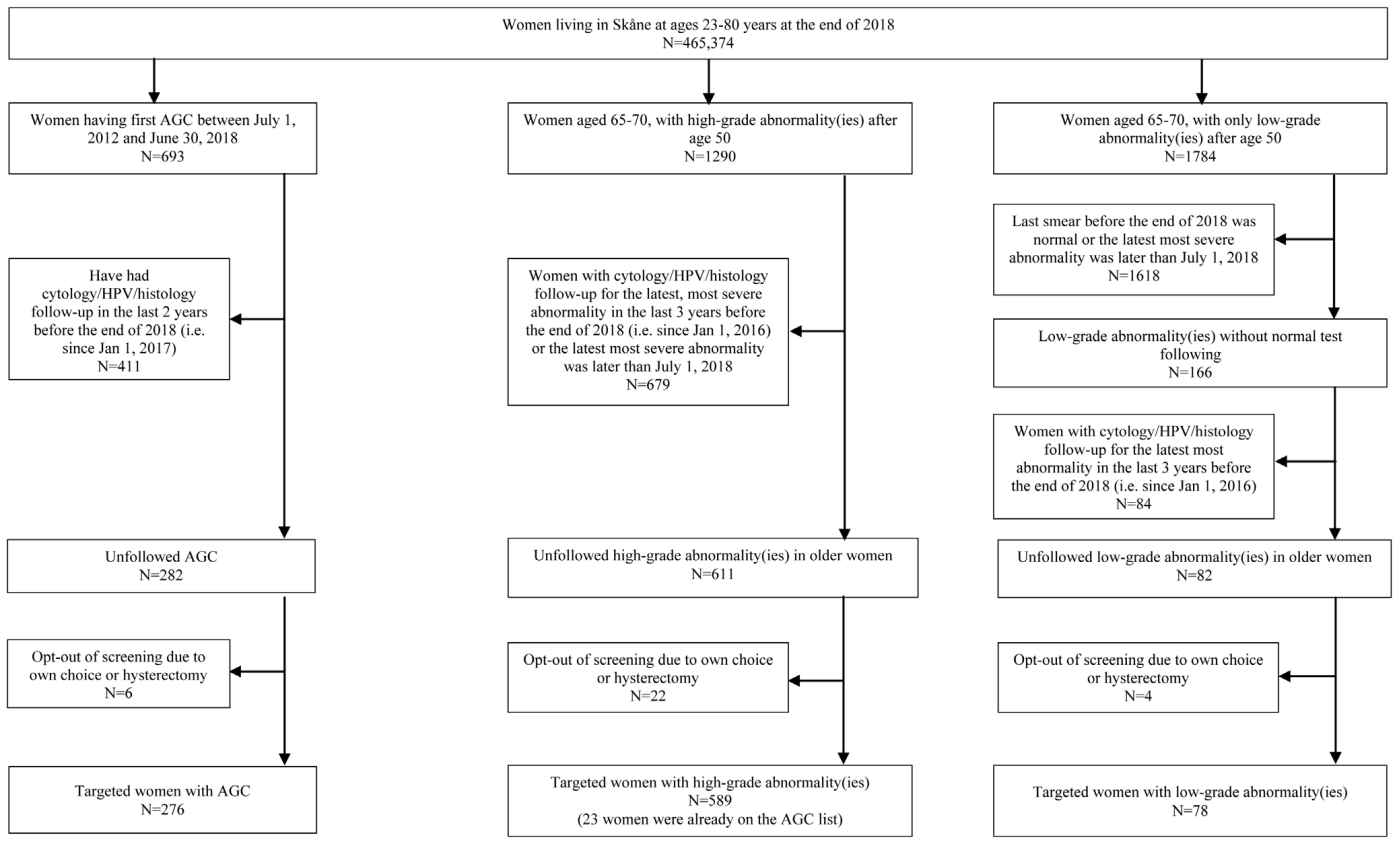
Flow-chart of inclusion and exclusion of study population.

This study targeted the women in the population that, based on data in the screening registry, had the highest risk of cervical cancer. The absolute risks of cervical cancer among the different risk groups in the population are presented in Table 1. The identification of these groups as being the groups at highest risk of cervical cancer is described in previous publications ^5, 6^. The present project describes the initial piloting of a risk-stratified screening strategy and is therefore targeting only rather small, very high-risk groups. Inclusion criteria of this pilot study were women 1) living in Southern Sweden (Skåne region) up to age 80 years at the end of 2018, with a cytological diagnosis of AGC in the past 0.5–6.5 years, and without a follow-up test on record (cytology, HPV or cervical histology test) in the past two years by the end of 2018; 2) living in Southern Sweden, aged 65–70 years at the end of 2018, with a high-grade abnormality after age 50 and without any follow-up test in the past three years, or having had low-grade abnormalities after age 50 and without any subsequent normal test result. We identified these women from the Swedish National Cervical Screening Registry (NKCx.se) and linked them to the Swedish Total Population Register, through the women’s unique Swedish personal identification number (PNR). Details of study inclusion and exclusion criteria are presented in Figure 1.

**Table 1.** Absolute risk of invasive cervical cancer by risk groups, identified using the cervical screening registry.

High-risk groups	Description	Absolute risk (Incidence rate per 100,000 person-years)
**Atypical glandular cells (AGC)**	Women having had AGC found in cytology in the past 6.5 years	~150/100,000 person-years ^a^
**Women aged 65–70**		
** History of high-grade** **abnormality**	Women having had high-grade abnormality ^b^ since age 50 years, but no biopsy.	~100/100,000 person-years ^c^
** History of low-grade** **abnormality**	Women having had low-grade abnormality ^d^ since age 50 years but no follow-up ^e^	~ 65/100,000 person-years ^c^
** Not screened**	Women not having any cervical test ^e^ since age 50 years	~30/100,000 person-years ^c^
**Long-term non-attenders at** **screening ages**	Women aged 30-64 and not having any cervical test ^e^ in the past 10 years	~25-30/100,000 person-years ^f^

a.  Wang *et al.* BMJ 2016 (DOI: 10.1136/bmj.i276) b.  Including cytological diagnosis of CIN2, CIN3, AGC, AIS, AUO (atypia of unknown origin) and invasive cancer in cytological screening c.  Wang *et al.* PLOS Medicine 2017 (DOI: 10.1371/journal.pmed.1002414) and related analyses d.  Including cytological diagnosis of ASCUS and CIN1 in cytological screening e.  Having no cytology, no HPV testing nor histology test f.  Wang *et al.* Acta Oncologica 2020 (DOI: 10.1080/0284186X.2020.1764095)

We have previously found that sending an invitation to order an HPV self-sampling kit is an efficient strategy for achieving increased participation at low costs ^12^. We sent SMS to women who fulfilled the inclusion criteria and had a registered cellphone number. The message stated that they were invited to a free HPV self-sampling test, organized by Karolinska Institutet and Region Skåne. They were invited to read more about the study, provide consent, and order the test kit for home delivery via our kit ordering platform ( https://www.hpvcenter.se/kit/index.php) ^13^. On the platform, women could place the order by typing in their PNR. Only the women whose PNR was included in the project database could order a kit, and only one kit per PNR was permitted by the platform. The SMS invitation was sent on August 19, 2019. Two SMS reminders, on September 16 and October 7, 2019, were sent to the women who had not ordered a kit by these dates. In December 2019, a physical letter was sent to the home addresses of women who did not have a registered cellphone number or who had received three SMS but had still not placed an order.

We sent cervical HPV self-sampling kits (cobas ^®^ PCR Female Swab Sample Packet) to the registered home address of women who either i) placed an order on the web platform, or ii) called the contact phone number of the project written on the web platform. Women who received a kit performed vaginal self-sampling following step-by-step picture-based instructions ^12, 13^, and sent the sample back in a pre-paid return envelope included in the package. The samples were sent to the Center of Cervical Cancer Prevention at Karolinska University Hospital in Stockholm, and tested on the cobas® 4800 HPV Test platform (Roche Molecular Systems, Inc, South Branchburg, NJ) for HPV 16, 18, 31, 33, 35, 39, 45, 51, 52, 56, 58, 59, 66 and 68, as high-risk HPV genotypes (hrHPV). The result was reported as being positive or negative for HPV 16, HPV 18, or “other” hrHPV types. Samples that were tested positive for “other” high-risk HPV were further genotyped by Luminex ^14, 15^. Further details of ordering platform, kit, and HPV testing platform have been described in a previous randomized study ^12^. Women who tested positive for any of the targeted HPV types were contacted by the gynecologist who was regionally responsible for cervical screening (CB) for diagnostic workup, including HPV mRNA test, cytology test, colposcopy, and biopsy.

We evaluated this pilot study of risk-stratified screening by measuring the participation (defined as the number and proportion of kits ordered and returned), the proportion of HPV positivity among returned samples, and the number of cervical intraepithelial neoplasia grade 2 or above (CIN2+) diagnoses in histopathology. These measures were compared between type of screening history (AGC or abnormalities at older ages) and age groups, using chi-square or fisher’s exact tests. SAS version 9.4 (SAS Institute, Cary, NC) is used for data management and analysis. Statistical analyses used two-sided tests and P-value <0.05 as the significance limit.

Ethical approval of this project was granted by the Swedish Ethical Review Authority (Decision number DNR 2019–03166). The website homepage provided the patient information and possibility to electronically provide Informed consent. This was required before being able to order self-sampling kits. The consent included permission to manage and analyse personal data and the biological specimens. The broader project of SMS-based Summons in Cervical Screening in Sweden is registered on ClinicalTrials.gov with Identifier NCT04061967 (20 ^th^ August 2019).

## Results

We identified 920 women having had AGC, or had an abnormal screening result after age 50, without sufficient follow-up. The details of inclusions are presented in Figure 1.

**Figure 2.**
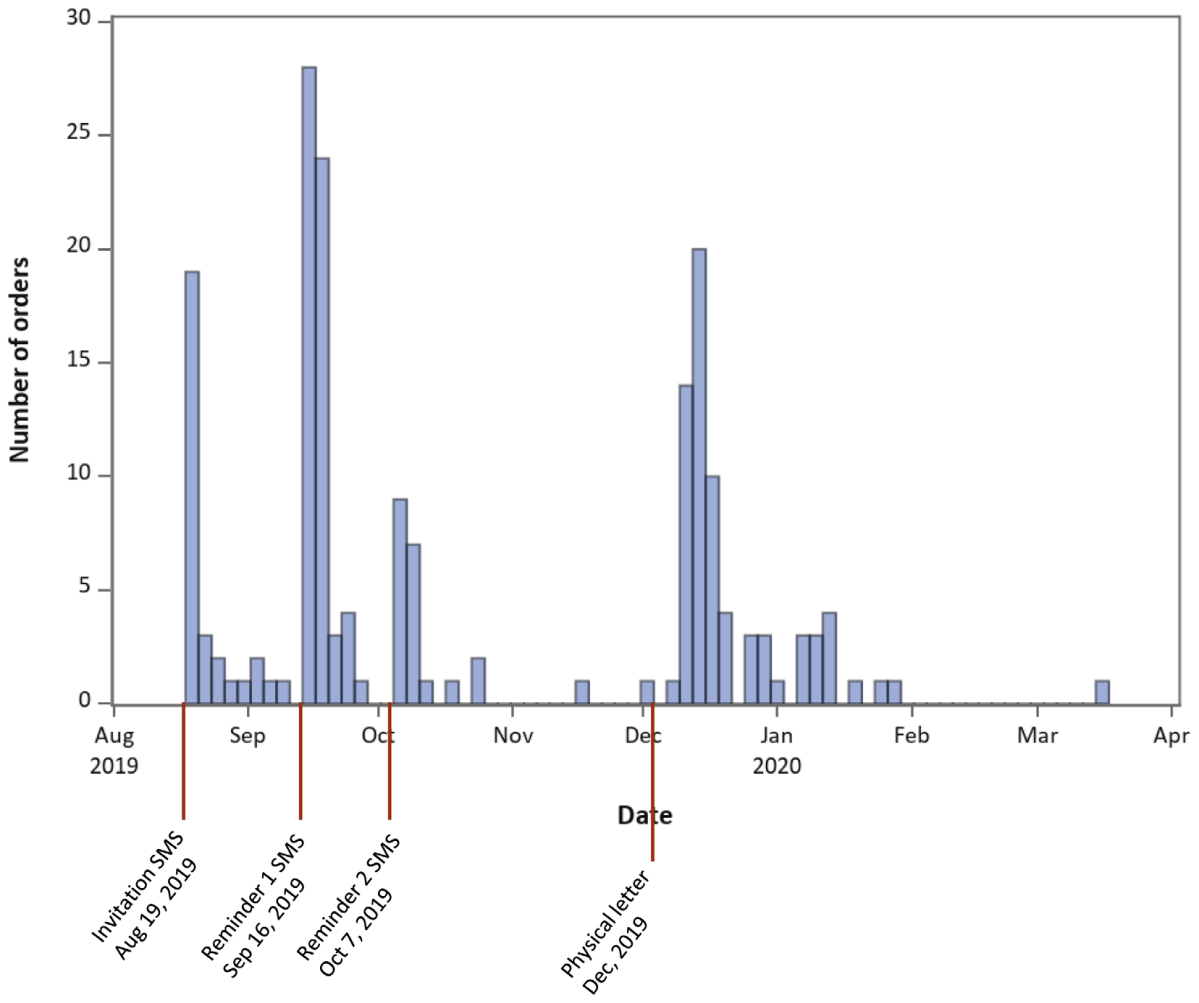
Number of kits ordered over time. Each bar represents 3 days.

Among 920 women, 531 had a registered cellphone number and hence were sent invitations and reminders through SMS. Non-responders after two reminders, as well as 389 women without a registered cellphone number were contacted through physical letters. Among all 920 women, 24 requested to be excluded, and 191 (20.8%) ordered the HPV self-sampling kit on the platform or through calling the contact phone number of the study. The orders were concentrated right after the invitation SMS was sent, the SMS reminders and the physical letter ( Figure 2). The orders increased in particular after sending the second SMS ( Table 2).

**Table 2.** Number of messages sent and orders by round and type of message.

	No. of messages sent	No. of orders * (%)
SMS invitation	531	30 (5.6)
SMS reminder 1	501	61 (12.2)
SMS reminder 2	440	21 (4.8)
Physical letter	808	70 (8.7)

*Nine women ordered through calling the contact phone number. Date of the order was not recorded.

Among 191 women who placed an order, 163 sent the sample back for HPV testing. The HPV testing results were as follows, 19 samples were HPV positive (11.7% out of tested), eight were HPV16/18, 10 were other hrHPV types including 31, 33, 51 and 56 ( Table 3). Nearly all women that tested positive attended the clinical follow-up and diagnostic work up (18 out of the 19 HPV-positive women). Eleven tested positive for HPV mRNA, seven had no lesions, two had low-grade lesions, and one had a CIN2+ diagnosis (Adenocarcinoma *in situ*, a 36-year-old woman who had had AGC before). The clinical follow-up was inconclusive for eight women, where a biopsy could not successfully be taken mostly because of cervical stenosis.

**Table 3.** Study population, participant and HPV positivity.

	No. of women	No. of drop-off	No. of ordered (%)	No. of tested (%)	No. of hrHPV+ve (% out of tested)	No. of HPV 16/18 positive	Genotypes of other hrHPV+ve				
							31	33	51	56	Negative
Total	920	24	191 (20.8)	163 (17.7)	19 (11.7)	8	3	1	4	2	1 ***
By age group											
23-40	47	2	5 (10.6)	3 (6.4)	1 (33.3)	1					
41-64	144	4	38 (26.4)	30 (20.8)	3 (10.0)	2	1				
65-70	673	16	139 (20.7)	121 (18.0)	14 (11.6)	5	1	1	4	2	1
71+	56	2	9 (16.1)	9 (16.1)	1 (11.1)	0	1				
By screening history **											
AGC	276	8	54 (19.6)	44 (15.9)	6 (13.6) *	3	2				
High-grade abnormality	566	16	123 (21.7)	108 (19.1)	9 (8.3) *	4			4		
Low-grade abnormality	78	0	14 (18.0)	11 (14.1)	4 (36.4) *	1	1	1		1	

* Statistically significant on two-sided chi-square test P-value <0.05. **Based on cytological screening finding. HPV, human papillomavirus; hrHPV, high risk HPV; AGC, atypical glandular cells. ***One woman tested positive for Other HPV in the Cobas test, but this could not be confirmed in theHPV genotyping

The proportion of participating women tended to be slightly higher among those aged 41–75 years, but the difference was not statistically significant. The proportion HPV positivity was particularly high in women with a history of low-grade abnormalities ( Table 3, probability=0.0031, P=0.02).

## Discussion

This pilot project invited women to HPV self-sampling with previous abnormal screening results lacking sufficient follow-up. Sending invitations and reminders through SMS for ordering a self-sampling kit online, complemented by sending physical letters to women who did not have a registered cellphone number or did not respond to three SMS invitations, was feasible and resulted in an acceptable participation rate. Clinical follow-up reached almost all women who tested HPV positive.

The participation in this study was higher than in our previous randomized trial of offering self-sampling kits to long-term non-attenders, where 12.9% of women ordered a kit and 10.7% returned a sample for testing in the arm that was invited to order a kit ^12^. A likely reason is that previously we sent only one invitation and it was sent only by physical letter. SMS invitations with reminders may outperform the traditional invitation letter. With SMS invitations, kit ordering is simpler because one can directly click the link to the web platform, without copying the web address written on a paper letter. Weaknesses of the SMS invitation strategy are that it may not appear as formal as the physical letter because of limited information and no stamp of authority such as a letterhead. The limited number of orders right after the initial SMS invitation may reflect that women were not used to receiving such invitation-SMSes. However, orders increased after an SMS reminder, suggesting that trust in the message can be strengthened simply by sending reminders of the invitation.

A majority of the study population was aged above 65 years and managing the kit ordering through cellphone and internet may be challenging. Nevertheless, the proportion of kit ordering in the older age group targeted in this study was not considerably lower than the younger group. There were only nine women who ordered the kit through contacting the research study by phone call. All of them were aged above 65 years, suggesting that the alternative ordering method is important, especially among older populations.

Only 1/10 women with an adequate colposcopy had a CIN2+ lesion. This was fewer than expected since these women were expected to have a high risk of cervical cancer due to their previous abnormal screening findings. The reasons behind this may include that 1) there was overall improvement in the routine clinical follow-up and management of women with abnormalities, especially after our publications indicating the high risk of these women, and with HPV testing becoming widely available. Under this circumstance, women with historical abnormality but without frequent follow-up, as are included in this project, may be the group with relatively lower risk triaged by previous clinical follow-up, e.g. negative in reflex HPV test; and 2) majority of the study population were post-menopausal, which makes the colposcopic and histopathological evaluation more challenging. Several HPV-positive women had cervical stenosis, and therefore the colposcopy was considered inadequate. These women will be followed with continuous surveillance. Nonetheless, the prevalence of HPV positivity in this study is higher than the general population, especially among older ages ^16, 17^. Identifying them and keeping them under surveillance may help to find precancer lesions or early staged invasive cervical cancer in time.

Another study also reported acceptance of self-sampling HPV testing after treatment of CIN lesions ^18^. To further improve participation in the target population in our study, we could consider sending physical letters to remind women and provide more information regarding the necessity of close follow-up, especially with HPV testing, after previous abnormalities. To optimize sample returns after kit ordering, we will consider sending an SMS reminder and a new kit to women who placed an order but did not return the sample.

Among the 3 very high-risk groups targeted in this study, 2 of these groups are women with insufficient follow-up after abnormal smears. Our assumption is that these women represent a group with non-attendance to follow-up, but the alternative explanation that management guidelines have not been followed cannot be excluded. Regardless of the explanation, the risk is high and targeting these women with risk-stratified screening is therefore motivated. For future strategies, much larger groups are planned to be targeted. A limitation with basing the identification of risk groups on the screening registry is that risk determinants that are not registered are not included, for example immunosuppressed women such as women living with HIV. The possibility to obtain not registered information on risk by asking the women will be explored. There were different HPV prevalences in the different risk groups, but as numbers are limited and biases by e.g. self-selection are possible caution is advised in interpreting these different HPV prevalences. An important outcome of the present pilot is that the response rates and HPV prevalences found will enable a cost-effectiveness evaluation of risk-stratified strategies that are planned for the future. 

As a pilot of risk-stratified cervical cancer screening, this study explored several procedures including the identification of certain risk groups from a well-established register database; efficient invitation strategies; systems to order, distribute and collect self-testing tools/samples; and clinical follow-up according to test results. Further efforts to improve risk-stratified screening strategies should be directed to improving the risk stratification algorithm; to developing an integrated system for invitation, test ordering and dispensing, sample collecting and testing, as well as result registration and delivery.

## Conclusion

This pilot study demonstrates that offering self-sampling HPV tests to high-risk women is feasible. We envision that such catch-up screening for high-risk groups could be performed in a whole country, and be a regular process complementing the routine screening program.

## Data availability

### Underlying data

B2SHARE: A pilot study of risk-stratified cervical cancer screening. http://doi.org/10.23728/b2share.10c44a0fe6f84f05b8f3c88b5681a4fc ^13^.

This project contains the following underlying data:

- datashare_JW20210423.xlsx (data underlying the study results)

### Extended data

B2SHARE: A pilot study of risk-stratified cervical cancer screening. http://doi.org/10.23728/b2share.10c44a0fe6f84f05b8f3c88b5681a4fc ^13^.

This project contains the following extended data:

- SMS invitation translation_JD20210524.docx (SMS invitation and reminder messages in English and Swedish).
- Step_by_step_instruction.pdf (step-by-step sampling instructions provided to participants)

Data are available under the terms of the Creative Commons Attribution-NonCommercial-NoDerivatives 4.0 International license (CC BY-NC-ND 4.0).
