# Supplementary material for: A pilot study of risk-stratified cervical cancer screening
Source: Open Res Eur. 2022 Sep 1;1:84. Originally published 2021 Jul 27. [Version 2] doi: 10.12688/openreseurope.13398.2 (PMC10445943; doi:10.12688/openreseurope.13398.2)
Supplement: Peer review history

# Peer review: Reviewer response for version 2

**DOI:** 10.21956/openreseurope.16261.r30010

**Published:** 2022-09-09

**Authors:** Raúl Murillo

**Affiliations:** 1 Centro Javerinao de Oncología, Hospital Universitario San Ignacio, Bogotá, Colombia

I think the authors fairly responded to my comments, so I would not have additional comments. Anyway, I am surprised that the authors do not have information on the sociodemographic characteristics of the patients despite stating that their study is based on a well-established registry, I think this is restricting a proper data analysis.

Is the study design appropriate and does the work have academic merit?

Partly

Is the work clearly and accurately presented and does it cite the current literature?

Partly

If applicable, is the statistical analysis and its interpretation appropriate?

Partly

Are all the source data underlying the results available to ensure full reproducibility?

Yes

Are the conclusions drawn adequately supported by the results?

Yes

Are sufficient details of methods and analysis provided to allow replication by others?

Yes

Reviewer Expertise:

Cancer early detection

I confirm that I have read this submission and believe that I have an appropriate level of expertise to confirm that it is of an acceptable scientific standard.

# Peer review: Reviewer response for version 2

**DOI:** 10.21956/openreseurope.16261.r30009

**Published:** 2022-09-02

**Authors:** Jean-Luc PRETET

**Affiliations:** 1 Centre National de Référence Papillomavirus, CHU Besançon, France, Besançon, France

I would like to thank the authors for their reponses to my comments and the modifications done on the manuscript.

Is the study design appropriate and does the work have academic merit?

Partly

Is the work clearly and accurately presented and does it cite the current literature?

Yes

If applicable, is the statistical analysis and its interpretation appropriate?

Partly

Are all the source data underlying the results available to ensure full reproducibility?

Yes

Are the conclusions drawn adequately supported by the results?

Partly

Are sufficient details of methods and analysis provided to allow replication by others?

Yes

Reviewer Expertise:

HPV-associated diseases, cervical cancer screening, HPV oncogenesis

I confirm that I have read this submission and believe that I have an appropriate level of expertise to confirm that it is of an acceptable scientific standard.

# Peer review: Reviewer response for version 1

**DOI:** 10.21956/openreseurope.14468.r27948

**Published:** 2021-11-18

**Authors:** Jean-Luc PRETET

**Affiliations:** 1 Centre National de Référence Papillomavirus, CHU Besançon, France, Besançon, France

The study by Wang and collaborators is a pilot project to test the feasibility and acceptability of offering a vaginal self-sampling for HPV testing in women with a high risk of cervical cancer. This pilot project is part of a larger one entitled RISCC (Risk-stratified Screening for Cervical Cancer). Women from a southern Sweden region (Skane region) with a high risk of cervical cancer were identified from the Swedish National Cervical Screening Registry according to two criteria: (i) women who had had an AGUS cytology during the past six years and (i) women above 50 years who had had an abnormal cytology but with an insufficient follow-up. These women were contacted by SMS and invited to order an HPV self-sampling kit on a dedicated internet platform. Two additional SMS were sent in the case the women did not respond and finally a physical invitation letter was sent after the two reminders by SMS if no order had been placed. A physical invitation letter was also sent to the women who did not have a registered cell phone number in the database. The main conclusions are that SMS invitations are feasible and resulted in an acceptable response rate. Furthermore, women who tested HPV positive were adequately followed-up.

 This is an interesting pilot project because it targets specific populations of women at high risk of cervical cancer. From a public health point of view, reaching these populations is important because they are certainly the ones who will benefit the most from a proactive cervical cancer screening program.

 Some points deserves to be highlighted and discussed.

 According to the methodology used it is difficult to determine whether the acceptable response rate is due to the SMS invitations or to the invitation letters. This is especially true after the last reminder by a physical letter (sent on Dec, 2019) since women who responded were either receiving the letter because they could not be reached by SMS or because they did not respond to the 3 SMS. I think that it could be interesting to distinguish in the figure 2 these two groups using stacked bar chart for example. The results then should be interpreted and discussed accordingly. As well, the conclusion paragraph in the abstract should be adapted because it is stated that “SMS invitations are feasible and result in substantial request”; invitation letters are not mentioned.

 The authors focused on two groups of “at risk” women. What about other “at risk” groups of women (immunosuppressed, HIV…). This was partly discussed when authors compared the results of the pilot study with those obtained with long-term non-attenders.

 One important point deals with the age of the study population and the authors pointed out that these women may not be comfortable with cell phone and internet. Can other means of ordering/picking-up self-sampling kits be considered (general practitioners, pharmacists, associations…).

 Does a cost-effectiveness study is planned? If not this should be at least discussed since cost-effectiveness analysis will help policy makers to take decision to implement public-health programs.

 Some minor points should also be addressed:

- page 4 first §: Can you indicate the name of test used to analyse genotypes by Luminex?
- page 4 second §: “kits” not “kids”?
- page 4 last §: 19 samples were HPV positive, with eight samples HPV16/18 and ten other HR HPV. One sample is lacking. Also clarify the “Total line” in the table 2.
- page 5 first §: “low-grade lesions” not “low-risk lesions”?
- page 5 second §: the proportion of HPV positivity in women with a history of abnormal cytology is high, but the number of women tested is very low.
- Figure 2: each bar seems to represent 3 days, is this correct?

Is the study design appropriate and does the work have academic merit?

Partly

Is the work clearly and accurately presented and does it cite the current literature?

Yes

If applicable, is the statistical analysis and its interpretation appropriate?

Partly

Are all the source data underlying the results available to ensure full reproducibility?

Yes

Are the conclusions drawn adequately supported by the results?

Partly

Are sufficient details of methods and analysis provided to allow replication by others?

Yes

Reviewer Expertise:

HPV-associated diseases, cervical cancer screening, HPV oncogenesis

I confirm that I have read this submission and believe that I have an appropriate level of expertise to confirm that it is of an acceptable scientific standard, however I have significant reservations, as outlined above.

# Response

**Published:** 2022-08-16

**Authors:** Jiangrong Wang

**Affiliations:** Karolinska Institute, Sweden

This is an interesting pilot project because it targets specific populations of women at high risk of cervical cancer. From a public health point of view, reaching these populations is important because they are certainly the ones who will benefit the most from a proactive cervical cancer screening program.

*Repl* *y:* *We are grateful for these supportive comments. *  

 Some points deserves to be highlighted and discussed. According to the methodology used it is difficult to determine whether the acceptable response rate is due to the SMS invitations or to the invitation letters. This is especially true after the last reminder by a physical letter (sent on Dec, 2019) since women who responded were either receiving the letter because they could not be reached by SMS or because they did not respond to the 3 SMS. I think that it could be interesting to distinguish in the figure 2 these two groups using stacked bar chart for example. The results then should be interpreted and discussed accordingly. As well, the conclusion paragraph in the abstract should be adapted because it is stated that “SMS invitations are feasible and result in substantial request”; invitation letters are not mentioned. *Repl* *y:* *This would have been interesting, but this exact data was not available (not registered because it was not specified in the ethical permission) and this subsplitting would have resulted in small groups.  For future projects on advancing risk-stratified screening, we will include this in the projects.* *We have revised the conclusion in the abstract adding the role of physical letter.*  

 The authors focused on two groups of “at risk” women. What about other “at risk” groups of women (immunosuppressed, HIV…). This was partly discussed when authors compared the results of the pilot study with those obtained with long-term non-attenders.

*Repl* *y:* *We discussed that women at high risk of cervical cancer can be readily identified by ordinary screening databases. The groups mentioned by the reviewer (HIV-positive and immunosuppressed women) would not be routinely registered in cervical screening databases and would be difficult to identify by registry linkages. This is now discussed in an added paragraph in the discussion section.*  

 One important point deals with the age of the study population and the authors pointed out that these women may not be comfortable with cell phone and internet. Can other means of ordering/picking-up self-sampling kits be considered (general practitioners, pharmacists, associations…).

*Repl* *y:* *As we discussed in the third paragraph of the Discussion section, the proportion of kit ordering in the older age group targeted in this study was not significantly lower than in the younger group.* *Strategies using pick-up of self-sampling kits are difficult to target for the small group of very high risk women targeted in this study. *  

 Does a cost-effectiveness study is planned? If not this should be at least discussed since cost-effectiveness analysis will help policy makers to take decision to implement public-health programs.

*Repl* *y:* *We have added a sentence about this in the discussion, the message being that such calculations cannot be done before data from a pilot on the response rate is available.*

 Some minor points should also be addressed: page 4 first §: Can you indicate the name of test used to analyse genotypes by Luminex?

*Repl* *y:* *It is an in-house test and the references to the detailed method is now given.*  

 page 4 second §: “kits” not “kids”?

*Repl* *y:* *Corrected.*  

 page 4 last §: 19 samples were HPV positive, with eight samples HPV16/18 and ten other HR HPV. One sample is lacking. Also clarify the “Total line” in table 2.

*Repl* *y:* *Indeed, there was one sample that had tested positive for “Other HPV” in the Cobas test, where the positivity could not be confirmed by Luminex. This is now noted in the Table.*

 page 5 first §: “low-grade lesions” not “low-risk lesions”? *Repl* *y:* *Corrected.* 

 page 5 second §: the proportion of HPV positivity in women with a history of abnormal cytology is high, but the number of women tested is very low.

*Repl* *y:* *We have pointed out that the paper describes a pilot study with a limited number of women.*  

 Figure 2: each bar seems to represent 3 days, is this correct?

*Repl* *y:* *Correct. Now described in the Figure legend.*

# Peer review: Reviewer response for version 1

**DOI:** 10.21956/openreseurope.14468.r27461

**Published:** 2021-09-14

**Authors:** Raúl Murillo

**Affiliations:** 1 Centro Javerinao de Oncología, Hospital Universitario San Ignacio, Bogotá, Colombia

Risk stratified screening is an innovative approach to find a more cost-effective way to implement population-based screening, and its main characteristic is the use of different screening protocols and algorithms for different subgroups within the target population according to the differential risk. Thus, risk stratified screening is based on risk, not on test results.

 In that sense, despite relevance, I consider the proposal by the authors actually an intervention for follow-up of non-adherent women with abnormal screening results rather than risk stratified screening as claimed in the title. Nonetheless, the lack of adherence could be *per se* a risk factor for developing CIN3+, but in my opinion, a proper risk approach should result in characterizing the non-adherent populations as part of the basis for risk estimates and contrasting screening modalities for the different risk categories identified.

 Still, the risk stratification implicit in the analysis deserve some clarification since it was defined based on the date of analysis instead the date of test results (diagnosis):

- The first group corresponds to women aged up to 80 years old with diagnosis of glandular atypia within the previous 0.5 to 6.5 years, but having no follow-up in the previous 2 years (ending on 2018). In my understanding, a given woman could have a diagnosis of glandular atypia 5 to 6 years ago with proper follow-up and negative results during 2 years after diagnosis, but according to the definition she would be at high risk because she does not have follow-up within the two years previous to the analysis.
- Similarly, the second group corresponds to women aged 65 to 70 years old with HSIL diagnosis after age 50 and no follow-up in the previous 3 years (ending on 2018). Again, a given woman could have an HSIL diagnosis 10 years ago (age 55) with proper follow-up and negative results during 5 years after diagnosis, but according to the definition she would be at high risk given the lack of records within the 3 years previous to the analysis.
- Finally, the third group, which would be from a clinical perspective the one with the lowest risk among the three, is defined based on the total lack of adherence regardless the time since diagnosis.

In my view, a proper risk approach should be based on the elapsed time without follow-up since diagnosis: the longer the time the higher the risk. As the study design apparently did not take this condition into account, I kindly suggest to include it in the analysis either in a prospective approach, ideal in my opinion (time without follow-up on record after diagnosis), or in a retrospective approach (time since the last follow-up test on record). I believe this approach would allow a better selection of at risk population and would provide a more accurate idea of losses to follow-up. At present, according to the definitions provided there are 276 women without follow-up out of 693 women with AGC (39.8%), 589 women without follow-up out of 1290 women with HSIL (45.7%), and 78 women without follow-up out of 1784 with LSIL (4.4%). Data of the two former groups resemble non-organized programs from low and middle income countries rather than highly organized programs with good call and recall systems in place, and apparently, losses to follow-up are lower among the lower risk populations (LSIL) which would not be expected on regular basis either. However, for a better assessment by the reader a brief description of the screening algorithm in the program analyzed would be highly desirable: regular screening interval (women at an average risk and women returning to normal after AGC or HSIL management), as well as follow-up protocols after AGC, HSIL and LSIL findings.

 The risk stratification based on date of data analysis instead of date of diagnosis explain the finding of higher high-risk HPV prevalence among the LSIL population, a result not discussed by the authors. Since the definition for this category is based on the absolute absence of any normal follow-up, they actually represent the group with the highest risk. Similarly, the finding of low CIN2+ prevalence contradicts the high-risk definition. On this regard, the authors hypothesized an improvement in routine follow-up as the main reason but they are defining risk precisely based on lack of follow-up, which is contradictory as indicated.

 In summary, I think the reported work better correspond to an acceptability study on HPV self-sampling among reluctant-to-follow-up women; although, I also think the acceptability analysis could be improved to get the most from the data. The characteristics of the study population are not reported regarding the basic approach: SMS vs postal mail. It is possible that women having no identified cell phone differ from those having cell phone in terms of age, income level, education level, and technology literacy. Such characteristics could be also related to the willingness to adopt new technologies making a difference between those who did and did not ordered the kit. Previous reports have shown that early and late technology adopters do not differ in age but they differ in income and education level, technology literacy, and social support and mobility (social networks). I am aware that not all variables of interest would be available in the data sources reported by the authors, but certainly some of them would be. I believe such kind of analysis would allow a better segmentation of the target population for planning future interventions.

 Regarding the intervention, the authors should take into account that one group had three reminders (two by SMS and one by postal mail), while the other group had only the initial invitation. This difference added to differences in baseline characteristics should be considered to properly interpret the results, and more relevant, the authors should not mix those receiving a postal mail as an additional reminder after two missing SMS with those having only the initial invitation by postal mail (physical letter). Indeed, analyzing the response rate after the additional reminder by postal mail would be worthy.

 Given the limitations described, interpreting the acceptability results is challenging. A 20.8% response rate to the invitation is low, but I am not certain about the risk of the population as previously noted. Having 18 out of 19 positive women attending the diagnostic workup is a very positive result, but as a reader I am not certain if those women are actually non-adherent (time without follow-up since diagnosis or time since the last follow-up test). In addition, I consider the clinical benefit uncertain not only because just one CIN2+ was found but also because 8 out of the 18 women attending the diagnostic workup had inconclusive results due to cervical stenosis, a condition associated with the age of the target population at risk as defined by the authors (women aged up to 80 years old). Thus, given the high frequency expected for such condition an alternative approach should be proposed otherwise unethical to keep the inclusion criteria.

 A basic question regarding acceptability of HPV self-collection is the consideration of this technology only for “high-risk” women versus an alternative for all women lost to follow-up, meaning even those without abnormal results but non-adherent to the regular screening interval. Although useful, if the latter the data of the present report should be ideally supplemented and contrasted with analyses on acceptability in the remaining groups of the target population. This contrast might be out of the scope of the publication, but a better characterization of the study population to allow such comparison with previously and future published literature is anticipated.

Is the study design appropriate and does the work have academic merit?

Partly

Is the work clearly and accurately presented and does it cite the current literature?

Partly

If applicable, is the statistical analysis and its interpretation appropriate?

Partly

Are all the source data underlying the results available to ensure full reproducibility?

Yes

Are the conclusions drawn adequately supported by the results?

Yes

Are sufficient details of methods and analysis provided to allow replication by others?

Yes

Reviewer Expertise:

Cancer early detection

I confirm that I have read this submission and believe that I have an appropriate level of expertise to confirm that it is of an acceptable scientific standard, however I have significant reservations, as outlined above.

# Response

**Published:** 2022-08-16

**Authors:** Jiangrong Wang

**Affiliations:** Karolinska Institute, Sweden

Risk stratified screening is an innovative approach to find a more cost-effective way to implement population-based screening, and its main characteristic is the use of different screening protocols and algorithms for different subgroups within the target population according to the differential risk. Thus, risk stratified screening is based on risk, not on test results. In that sense, despite relevance, I consider the proposal by the authors actually an intervention for follow-up of non-adherent women with abnormal screening results rather than risk stratified screening as claimed in the title. Nonetheless, the lack of adherence could be per se a risk factor for developing CIN3+, but in my opinion, a proper risk approach should result in characterizing the non-adherent populations as part of the basis for risk estimates and contrasting screening modalities for the different risk categories identified.

 Still, the risk stratification implicit in the analysis deserve some clarification since it was defined based on the date of analysis instead the date of test results (diagnosis): The first group corresponds to women aged up to 80 years old with diagnosis of glandular atypia within the previous 0.5 to 6.5 years, but having no follow-up in the previous 2 years (ending on 2018). In my understanding, a given woman could have a diagnosis of glandular atypia 5 to 6 years ago with proper follow-up and negative results during 2 years after diagnosis, but according to the definition she would be at high risk because she does not have follow-up within the two years previous to the analysis.   Similarly, the second group corresponds to women aged 65 to 70 years old with HSIL diagnosis after age 50 and no follow-up in the previous 3 years (ending on 2018). Again, a given woman could have an HSIL diagnosis 10 years ago (age 55) with proper follow-up and negative results during 5 years after diagnosis, but according to the definition she would be at high risk given the lack of records within the 3 years previous to the analysis.

 Finally, the third group, which would be from a clinical perspective the one with the lowest risk among the three, is defined based on the total lack of adherence regardless the time since diagnosis. In my view, a proper risk approach should be based on the elapsed time without follow-up since diagnosis: the longer the time the higher the risk. As the study design apparently did not take this condition into account, I kindly suggest to include it in the analysis either in a prospective approach, ideal in my opinion (time without follow-up on record after diagnosis), or in a retrospective approach (time since the last follow-up test on record). I believe this approach would allow a better selection of at risk population and would provide a more accurate idea of losses to follow-up. At present, according to the definitions provided there are 276 women without follow-up out of 693 women with AGC (39.8%), 589 women without follow-up out of 1290 women with HSIL (45.7%), and 78 women without follow-up out of 1784 with LSIL (4.4%). Data of the two former groups resemble non-organized programs from low and middle income countries rather than highly organized programs with good call and recall systems in place, and apparently, losses to follow-up are lower among the lower risk populations (LSIL) which would not be expected on regular basis either. However, for a better assessment by the reader a brief description of the screening algorithm in the program analyzed would be highly desirable: regular screening interval (women at an average risk and women returning to normal after AGC or HSIL management), as well as follow-up protocols after AGC, HSIL and LSIL findings.  

*Repl* *y:* *We are very grateful for these careful and detailed comments.* *Regarding only targeting women with previous abnormality in this risk-stratified screening pilot, we s* *imply targeted the women with highest risk in the population. The risk profile calculations were retrieved from previous publications, but to make the procedure more clear to the readers we added an extra Table with the exact algorithm and the exact cervical cancer risk of the targeted risk groups.* *We also added an extra description at the beginning of the Method section. Of course, future projects with risk stratified screening will use much larger risk groups.* *In response to the series of comments, we added explanations of the risk group identification in the discussion (see the added paragraph in the discussion section).*

 The risk stratification based on date of data analysis instead of date of diagnosis explain the finding of higher high-risk HPV prevalence among the LSIL population, a result not discussed by the authors. Since the definition for this category is based on the absolute absence of any normal follow-up, they actually represent the group with the highest risk. Similarly, the finding of low CIN2+ prevalence contradicts the high-risk definition. On this regard, the authors hypothesized an improvement in routine follow-up as the main reason but they are defining risk precisely based on lack of follow-up, which is contradictory as indicated.  

*Repl* *y:* *Indeed, an intervention to find women at high risk starts when the intervention is launched and identifies the women with risk profiles in their screening history. This is in contrast to the gynecological follow-up which starts when abnormalities are detected. As the study was not about optimal follow-up, but about risk-stratified screening, identification of risk women should start when the screening is launched.* *This is now clarified.* *On the difference in HPV prevalences between various groups, we did not want to speculate too much because of small numbers. Nevertheless, we added a brief explanation in the discussion section (see the added paragraph in discussion section).*

 In summary, I think the reported work better correspond to an acceptability study on HPV self-sampling among reluctant-to-follow-up women; although, I also think the acceptability analysis could be improved to get the most from the data. The characteristics of the study population are not reported regarding the basic approach: SMS vs postal mail. It is possible that women having no identified cell phone differ from those having cell phone in terms of age, income level, education level, and technology literacy. Such characteristics could be also related to the willingness to adopt new technologies making a difference between those who did and did not ordered the kit. Previous reports have shown that early and late technology adopters do not differ in age but they differ in income and education level, technology literacy, and social support and mobility (social networks). I am aware that not all variables of interest would be available in the data sources reported by the authors, but certainly some of them would be. I believe such kind of analysis would allow a better segmentation of the target population for planning future interventions.  

*Repl* *y:* *Comparting social- and demographic characteristics between women who responded to SMS and postal mail would have been interesting, but this data was unfortunately not available.*

 Regarding the intervention, the authors should take into account that one group had three reminders (two by SMS and one by postal mail), while the other group had only the initial invitation. This difference added to differences in baseline characteristics should be considered to properly interpret the results, and more relevant, the authors should not mix those receiving a postal mail as an additional reminder after two missing SMS with those having only the initial invitation by postal mail (physical letter). Indeed, analyzing the response rate after the additional reminder by postal mail would be worthy.

*Repl* *y:* *This is a valuable suggestion and we will certainly collect such data in future studies. It was, however, not collected in this study.*

 Given the limitations described, interpreting the acceptability results is challenging. A 20.8% response rate to the invitation is low, but I am not certain about the risk of the population as previously noted. Having 18 out of 19 positive women attending the diagnostic workup is a very positive result, but as a reader I am not certain if those women are actually non-adherent (time without follow-up since diagnosis or time since the last follow-up test). In addition, I consider the clinical benefit uncertain not only because just one CIN2+ was found but also because 8 out of the 18 women attending the diagnostic workup had inconclusive results due to cervical stenosis, a condition associated with the age of the target population at risk as defined by the authors (women aged up to 80 years old). Thus, given the high frequency expected for such condition an alternative approach should be proposed otherwise unethical to keep the inclusion criteria.

*Repl* *y:* *In the discussion (the second paragraph), we now compared this 20.8% response rate to similar trials among non-attenders (which was 10.7% under the same kit-order manner).* *In the discussion section, we have now underlined that women with inconclusive follow-up because of cervical stenosis will continue to be followed up. Indeed, it would have been unethical to just let these women go.*

 A basic question regarding acceptability of HPV self-collection is the consideration of this technology only for “high-risk” women versus an alternative for all women lost to follow-up, meaning even those without abnormal results but non-adherent to the regular screening interval. Although useful, if the latter the data of the present report should be ideally supplemented and contrasted with analyses on acceptability in the remaining groups of the target population. This contrast might be out of the scope of the publication, but a better characterization of the study population to allow such comparison with previously and future published literature is anticipated.

*Repl* *y:* *Indeed. We did compare the response rate in this pilot study with the previous trials among non-attenders. In the new Table we compare the cancer risk to the non-attenders without previous abnormal smears. Direct comparison with these long-term non-attenders without abnormal smears will be subject of future projects.*
